# The Schematic Assessment of Vitamin D Deficiency in Relation to Autoimmune Disorders and Its Implications in Internal Medicine

**DOI:** 10.7759/cureus.82949

**Published:** 2025-04-24

**Authors:** Junaid Aslam, Mohammad Sohailuddin, Syed M Abbas, Muhammad Tamoor A Shaikh, Shanza Saleem, Arooj Mubeen, Basil Ahmad, Muhammad Haseeb, Ehsan Ul Haq Mzahri

**Affiliations:** 1 Department of Internal Medicine, Lahore General Hospital, Postgraduate Medical Institute, Ameer-ud-Din Medical College, Lahore, PAK; 2 Department of Accident and Emergency, Maidstone and Tunbridge Wells Hospital, Maidstone, GBR; 3 Department of General Medicine, Bangor Hospital, Wwales, GBR; 4 Department of Cardiology, Chaudhary Pervaiz Elahi Institute of Cardiology, Wazirabad, PAK; 5 Department of General Medicine, Punjab Rangers Teaching Hospital, Lahore, PAK; 6 Intensive Care Unit, Hameed Latif Hospital, Lahore, PAK; 7 Department of Cardiology, Warrington and Halton Teaching Hospitals, NHS Foundation Trust, Warrington, GBR; 8 Department of Oncology, Shaikh Zayed Hospital, Lahore, PAK; 9 Department of Health Sciences and Pathology, University of the Punjab, Lahore, PAK; 10 Department of Pathology and Oncology, University of Health Sciences, Lahore, PAK; 11 Department of Pathology, University of Indonesia, Jakarta, IDN

**Keywords:** autoimmune diseases, disease activity, immune system, internal medicine, systematic review, vitamin d deficiency

## Abstract

The immune system greatly depends on vitamin D for modulating its function, despite the fact that deficiency of this nutrient is now well recognized as a factor that contributes to the development of several autoimmune disorders. This study observed the occurrence of vitamin D deficiency among autoimmune disease patients and investigated its implications in internal medicine. Two medical research investigators searched through several electronic databases such as PubMed, Scopus, Web of Science, and Google Scholar for studies ranging from 2018 to 2025. The research examined the findings of published studies that measured serum vitamin D levels of autoimmune disorder patients in conjunction with how this measurement affected disease activity, severity, and therapy results. A review process executed a predefined research eligibility framework and incorporated both observational and interventional study types for selection. The extraction of study data was done autonomously by two reviewers. Two tools were used to evaluate the methodological quality of the selected studies: the Newcastle-Ottawa Scale and the Cochrane Risk of Bias Tool. The strength of evidence was evaluated using the Grading of Recommendations, Assessment, Development and Evaluation (GRADE) framework. The research analysis included a total of eight studies that fulfilled the eligibility requirements. Most studies identified vitamin D deficiency as common among patients who suffered from rheumatoid arthritis, systemic lupus erythematosus, and multiple sclerosis. The literature showed increased severity of disease in patients with insufficient vitamin D levels. Some researchers also observed symptom improvement following vitamin D supplementation. Overall, the reviewed evidence suggested that vitamin D may be a potentially modifiable element in the management of autoimmune diseases. While research demonstrates a connection between vitamin D deficiency and autoimmune disease evolution, additional clinical testing must confirm the usefulness of vitamin D therapy in internal medicine.

## Introduction and background

Recent scientific evidence demonstrates that, alongside its essential role in bone health, vitamin D serves as a fundamental agent for regulating the immune system [1]. The increasing incidence of different autoimmune disorders has been linked to deficient levels of vitamin D [2]. Research has confirmed a strong connection between reduced vitamin D levels in individuals with rheumatoid arthritis, multiple sclerosis, systemic lupus erythematosus, and type 1 diabetes [3-5]. Despite advancements in internal medicine and therapeutic approaches, the difficulties caused by autoimmune diseases have not been eliminated due to their complex pathophysiology and unpredictable course [6]. Identifying new risk factors for autoimmune disorder management remains crucial, with vitamin D now recognized as a significant contributor to autoimmune disorders [7].

The main healthcare strategy uses immunosuppressive medicines to reduce symptoms, although these therapies do not specifically target nutritional elements or environmental influences that might trigger or worsen disease activity [8]. Research continues into vitamin D's immunomodulatory effects due to its potential value in developing therapeutic measures. Studies reveal that vitamin D controls different immune system responses, such as managing T cell activity, decreasing inflammatory protein expression, and supporting immune tolerance [9]. Vitamin D shows such attributes that suggest that it helps in preventing disease development while also boosting the effect of current treatments and improving patients' overall disease control [10].

The review methodically examined how vitamin D deficiency affects autoimmune diseases through its specific applications in internal medicine. The study analyzed the recent discoveries regarding how vitamin D levels affect disease activity, severity, and treatment responses for developing better clinical practices and patient healthcare.

## Review

Methodology

The analysis followed the Preferred Reporting Items for Systematic reviews and Meta-Analyses (PRISMA) 2020 guidelines to ensure both transparency and comprehensive evaluation of the relationship between vitamin D deficiency and autoimmune disorder in internal medicine settings. Included studies met predetermined eligibility criteria that evaluated both study design and research approach. The selected studies involved patients with autoimmune disorders and examined how vitamin D concentrations influenced disease activity, together with their clinical course and therapeutic behavior. The systematic search was conducted across multiple online databases, including PubMed, Scopus, Web of Science, and Google Scholar. The research focused on English-language literature published from 2018 to 2025. The search strategy combined terms such as "vitamin D deficiency" with "autoimmune diseases" and "immune regulation" and "internal medicine" with "disease activity." The search refinement was achieved through the utilization of Boolean operators and applying relevant filters to narrow down the results

The analyzed literature employed three primary search phrases: "vitamin D and autoimmunity", "vitamin D in internal medicine", and "immunomodulatory effects of vitamin D". A preliminary screening of titles and abstracts was used to identify articles for full-text review to assess relevance. Two independent experts evaluated the eligible studies, and any disagreements during assessment were settled either through team discussion or by consulting another expert. The reviewers used standardized data collection forms to obtain information and extract study features, patient cohorts, autoimmune conditions, vitamin D assessment findings, and documented clinical outcomes, independently from each other. A systematic evaluation of risk of bias was conducted for all included studies using appropriate tools such as the Newcastle-Ottawa Scale for observational investigations and the Cochrane Risk of Bias tool for randomized controlled trials. Due to variability in study designs, populations, and outcome measurements, a meta-analysis could not be performed. Instead, the findings were organized using a narrative synthesis, categorizing results by autoimmune disease, vitamin D deficiency severity, and observed clinical effects.

This study applied Grading of Recommendations, Assessment, Development and Evaluation (GRADE) assessments to evaluate the evidence based on study design, bias risk, and result consistency and directness. The organized research approach provided an appropriate framework to thoroughly analyze recent findings on the effects of vitamin D deficiency on autoimmune diseases while also identifying potential new.

Results

The review process resulted in the selection of 10 publications out of 104 academic manuscripts accessed through diverse electronic databases. Studies included in this research investigated how vitamin D deficiency affects autoimmune disorders at different stages, including initial development, disease progression, and treatment response. Among the selected studies, the researchers identified several observational designs, including six cross-sectional studies, one prospective open-label study, one randomized clinical trial, one case study, and one clinical trial.

**Figure 1:**
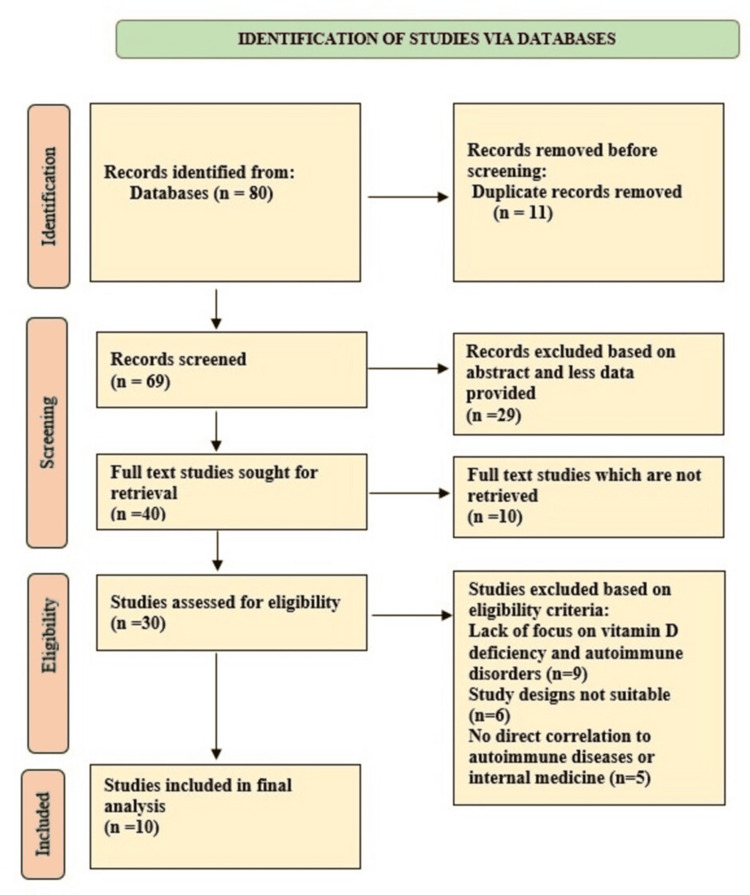
Filtration process of gathered studies till final selection of studies using PRISMA guidelines PRISMA - Preferred Reporting Items for Systematic reviews and Meta-Analyses

The research included studies with varying population sizes, ranging from 6 to 5230 participants, with a median of 50. Multiple autoimmune diseases were examined in this study, including rheumatoid arthritis, systemic lupus erythematosus, multiple sclerosis, type 1 diabetes mellitus, inflammatory bowel disease, psoriasis, Hashimoto's thyroiditis, and vitiligo. Figure 1 demonstrates the details of the study selection process below. 

The reviewed studies evaluated serum vitamin D levels, which serve as essential biochemical markers within rheumatoid arthritis, multiple sclerosis, systemic lupus erythematosus, and several other autoimmune disorders. Various research papers highlight vitamin D as an immunomodulator, influencing both inflammatory processes and autoantibody production, and impacting the natural progression of autoimmune diseases. The review also uncovered additional insights that are not present in the original studies, such as the influence of seasonal vitamin D variations on flare frequencies. Medical research suggests that vitamin D deficiency is linked to elevated disease activity, more frequent relapses, and unfavorable clinical results in several autoimmune diseases, as shown in Table 1.

**Table 1:** Systematic review table showcasing characteristics and key findings of individual studies SLEDAI - Systemic Lupus Erythematosus Disease Activity Index; PASI - Psoriasis Area and Severity Index; BMD - bone mineral health; VETI - Vitiligo Extent Tensity Index

Study details	Sample size	Study design	Autoimmune disease	Outcomes	Key findings
Tv et al., 2023 (India) [11]	50	Cross-sectional	Rheumatoid arthritis	The vitamin D deficiency was seen in 50% of the patients	Vitamin D deficiency is significantly correlated with the disease severity of rheumatoid arthritis.
Magro et al., 2021 (Malta) [12]	31	Prospective open-label	Systemic lupus erythematosus	Vitamin D3 supplementation improved disease activity (SLEDAI, p=0.028), reduced anti-dsDNA (p=0.045), and showed a trend in fatigue improvement (p=0.071).	Lower vitamin D levels are linked with increased anti-dsDNA antibody titres.
Thouvenot et al., 2025 (France) [13]	303	Randomized clinical trial	Multiple sclerosis	Vitamin D3 significantly reduced relapse/MRI activity over 24 months (HR=0.66, p<0.05)	Patients with low vitamin D had more frequent relapses and MRI activity.
Dehkordi et al., 2025 (Iran) [14]	30	Cross-sectional	Type 1 diabetes mellitus	Vitamin D3 supplementation significantly increased serum vitamin D (p<0.001)	Reduced vitamin D levels are associated with poor glycemic control.
Jalili et al., 2019 (Norway) [15]	116	Clinical trial	Inflammatory bowel disease (IBS)	BS-TS improved in S+P and D+P groups (p=0.004, 0.015), with a significant interaction effect on IBS-TS (p<0.05), while IBS-SSS decreased in S+P and D+P groups (p=0.001, 0.047)	Vitamin D supplementation significantly improved IBS symptoms severity, disease-specific QOL, and total score
Mahtani et al., 2022 (India) [16]	6	Case-control	Psoriasis	High-dose oral vitamin D3 (30,000–60,000 IU/day) achieved complete psoriasis control (PASI ≤0.8, VAS ≤1) in all 6 patients within 2-8 months without adverse effects.	Vitamin D deficiency is linked to disease severity. High doses of daily oral Vitamin D3 for 2-6 months resulted in complete control of psoriasis without adverse effects.
Chao et al., 2020 (China) [17]	5230	Cross-sectional	Hashimoto's thyroiditis (HT)	Lower 25(OH)D levels were associated with higher TSH and lower FT3/FT4, with each 1 ng/mL increase in 25(OH)D linked to a 2.78 ng/dL rise in FT4 and 0.17 mIU/L drop in TSH (n = 5,230).	HT patients had lower levels of Vitamin D compared to non-HT patients. TSH levels were higher in vitamin D insufficiency and deficiency groups.
Akelma et al., 2022 (Turkiye) [18]	6717	Cross-sectional	Celiac disease (CD)	Children with celiac disease had significantly lower median 25(OH)D levels (18.5 vs. 30.7 ng/mL) and higher deficiency rates (56% vs. 12%) compared to controls (p<0.001).	Vitamin D levels were significantly lower in children with CD compared to controls.
Kocyigit et al., 2018 (Turkiye) [19]	68	Cross-sectional	Ankylosing spondylitis	Ankylosing spondylitis patients had significantly lower vitamin D and BMD values (p≤0.011), while disease activity inversely correlated with BMD (p<0.05).	Vitamin D levels and BMD were lower in ankylosing spondylitis patients.
Mahmmod and Ismael et al., 2022 (Iraq) [20]	46	Cross-sectional	Vitiligo	Most vitiligo patients had low vitamin D levels (p<0.05), mostly females (p=0.642), with no significant effect on VETI scores (p=0.184).	The majority of vitiligo patients had low vitamin D levels, with higher deficiency observed in females.

Research by Padmapriya et al. (2023) and Magro et al. (2021) has provided consolidated evidence linking vitamin D status to autoimmune disease conditions. According to Padmapriya et al. (2023). Magro et al. (2021) found that patients with systemic lupus erythematosus (SLE) who had vitamin D deficiency experienced increased disease flares and elevated anti-dsDNA antibodies. The research by Hashemi Dehkordi et al. (2025) showed that poor glycemic control in patients with type 1 diabetes mellitus (T1DM) is strongly correlated with reduced vitamin D levels in the blood. Furthermore, Thouvenot et al. (2025) reported that patients with insufficient vitamin D exhibited elevated relapse rates and increased MRI activity when compared with those who maintained normal vitamin D levels [11-14]. Research by Jalili et al. (2019) demonstrated that vitamin D supplementation can enhance symptoms in irritable bowel syndrome (IBS) patients, leading to a better quality of life through decreased disease severity. Chao et al. (2020) demonstrated that insufficient vitamin D exists as a direct cause of Hashimoto's thyroiditis, showing reduced vitamin D levels alongside elevated thyroid-stimulating hormone (TSH) levels, which lead to thyroid dysfunction [15-17].

Most of the analyzed studies exhibited a moderate to high risk of bias. The risk in cohort studies was elevated due to their unrandomized methods, unclear procedures for allocation, and issues with participant enrollment. Assessment protocols within randomized controlled trials were unclear according to the Cochrane Risk of Bias Tool, as outcome data contained missing information, as shown in Table 2. Variations in bias control against confounding variables were observed in cohort studies, as noted by the Newcastle-Ottawa Scale, shown in Table 3. The reviewers engaged in ongoing discussion until they reached a consensus. The review team employed a third reviewer to settle disagreements only when required.

**Table 2:** Risk of bias assessment for randomized clinical trials (RCTs) "+" indicates a low risk of bias, "±" indicates an unclear or moderate risk of bias, and"-" indicates a high risk of bias

Study	Sequence generation (selection bias)	Allocation concealment (selection bias)	Blinding of participants and personnel (performance bias)	Blinding of outcome assessment (detection bias)	Incomplete outcome data	Selective outcome reporting	Other bias
Kocyigit et al., 2018 (Turkiye) [19]	±	±	±	+	+	±	±
Thouvenot et al., 2025 (France) [13]	+	+	+	+	+	+	±
Akelma et al., 2022 (Turkiye) [18]	±	+	+	+	±	+	±
Jalili et al., 2019 (Norway) [15]	+	+	+	+	+	+	+

**Table 3:** Risk of bias assessment for observational studies

Study	Selection (Max 4)	Comparability (Max 2)	Outcome (Max 3)	Total Score (Max 9)
Tv et al., 2023 (India) [11]	★★★	★	★★	6
Magro et al., 2021 (Malta) [12]	★★★	★★	★★	7
Dehkordi et al., 2025 (Iran) [14]	★★★★	★★	★★	8
Mahtani et al., 2022 (India) [16]	★★★	★	★★	6
Mahmmod & Ismael et al., 2022 (Iraq) [20]	★★	★	★★	5
Chao et al., 2020 (China) [17]	★★★	★	★★	6

Research on vitamin D treatment for autoimmune diseases yielded promising results according to assessment, yet the evidence remains at a moderate level. Various studies faced performance limitations due to small participant groups, insufficient prospective testing, and methodological shortcomings. Based on the GRADE assessment, most studies were insufficient to support the direct use of therapeutic vitamin D in clinical applications. The observed effects from these research studies need confirmation through extensive clinical testing across multiple facilities.

Discussion

The research examined the link between vitamin D status and biomarker evaluation in autoimmune disorders, focusing on their impact on disease progression and clinical outcomes. The research analyzed 10 crucial studies to determine if serum vitamin D levels accurately reflect autoimmune disease pathology. Research shows that vitamin D serves as both an effective tool for diagnosing and prognosing autoimmune disorders, as it affects immune function regulation and disease progression. A wide spectrum of autoimmune diseases, such as rheumatoid arthritis, systemic lupus erythematosus, multiple sclerosis, type 1 diabetes mellitus, and Hashimoto's thyroiditis, were included in the results. Many studies demonstrated that disease activity of patients worsened in patients with vitamin D deficiency who also exhibited an increased relapse risk alongside biomarker changes. These findings align with scientific understanding of T-cell regulation, cytokine signaling, and immune balance control by vitamin D [21]. The association between high disease severity, vitamin D insufficiency, and altered inflammatory markers supports its potential role as a diagnostic indicator of autoimmune disease treatment [22].

These encouraging observational trends face major barriers that prevent their utilization in medical practices. Analysis of vitamin D research becomes complex due to the conflicting definitions of vitamin D sufficiency, too few patient participants, and different amounts of sunlight in researched areas [23]. Standardized laboratory protocols need to be developed, and diagnostic thresholds need to match for researchers to achieve replicability [24].

Variations between research methods, study populations, and clinical end result measurements limit the application of these results to broader autoimmune patient groups. Research shows that vitamin D supplements show benefits for disease management, especially in psoriasis patients and those with irritable bowel syndrome [25]. Inadequate replication methods, along with minimal follow-up data, make it difficult to draw solid conclusions about the topic. Vitamin D levels seem to affect measures including TSH, thyroid peroxidase (TPO) antibodies, and neutrophil-to-lymphocyte ratio (NLR), which provides possible opportunities for individualized disease control [26, 27].

Current research shows that vitamin D interacts with both hereditary elements and molecular signaling networks, which affect the development path of immune disorders. Research indications demonstrate that vitamin D acts as a biomarker for prediction; however, inconsistencies appear in patient reactions, combined with variable dosage approaches and follow-up periods, which create knowledge gaps. Additional large-scale controlled research must be performed to confirm vitamin D's effectiveness for disease prediction assessment as well as its best clinical application methods.

## Conclusions

The review system validated the existence of vitamin D as a diagnostic biomarker, which helps professionals monitor autoimmune system disorders. A variety of conditions showed evidence that vitamin D deficiency leads to worsened disease progression and affects immune system functioning. The reported evidence indicates that vitamin D status reflects immune dysfunction levels; thus, it can assist medical decisions during autoimmune treatment management. 

The evaluated research showed inconsistent sample sizes, differences in demographic groups, and biomarker assessment approaches, which restricted the validity of their findings. Research using vitamin D as a biomarker needs to integrate additional patient-specific and disease variation analyses to produce effective results. Research on vitamin D should explore its relationship with genetic influences and molecular processes to improve forecasting abilities. Standardized measures of assessment combined with uniform trial methods serve as fundamental requirements for making vitamin D an operational biomarker for autoimmunity disease control in clinical settings.
